# Polymer Composites with Carbon Fillers Based on Coal Pitch and Petroleum Pitch Cokes: Structure, Electrical, Thermal, and Mechanical Properties

**DOI:** 10.3390/polym16060741

**Published:** 2024-03-08

**Authors:** Yevgen Mamunya, Andrii Misiura, Marcin Godzierz, Sławomira Pusz, Urszula Szeluga, Karolina Olszowska, Paweł S. Wróbel, Anna Hercog, Anastasiia Kobyliukh, Andrii Pylypenko

**Affiliations:** 1 Institute of Macromolecular Chemistry, NAS of Ukraine, 48, Kharkivske Chaussee, 02160 Kyiv, Ukraine; andrii_misiura@ukr.net (A.M.); pik70@ukr.net (A.P.); 2 Taras Shevchenko National University of Kyiv, 64, Volodymyrska St., 01033 Kyiv, Ukraine; 3 Centre of Polymer and Carbon Materials, Polish Academy of Sciences, M. Curie-Skłodowskiej 34, 41-819 Zabrze, Poland; spusz@cmpw-pan.pl (S.P.); uszeluga@cmpw-pan.pl (U.S.); kolszowska@cmpw-pan.pl (K.O.); pwrobel@cmpw-pan.pl (P.S.W.); ahercog@cmpw-pan.pl (A.H.); akobyliukh@cmpw-pan.pl (A.K.)

**Keywords:** novel carbon filler, coke-based particles, segregated composite structure, electrical conductivity, mechanical properties

## Abstract

The effect of particle size and oxidation degree of new carbon microfillers, based on coal pitch (CP) and petroleum pitch (PET) cokes, on the structure as well as thermal, mechanical, and electrical properties of the composites based on ultrahigh molecular weight polyethylene (UHMWPE) was investigated. The composites studied have a segregated structure of filler particle distribution in the UHMWPE matrix. It was found that composite with smaller CP grain fraction has the highest Young’s modulus and electrical conductivity compared to the other composites studied, which can be the result of a large contribution of flake-shaped particles. Additionally, conductivity of this composite turned out to be similar to composites with well-known carbon nanofillers, such as graphene, carbon black, and CNTs. Additionally, the relationship between electrical conductivity and Young’s modulus values of composites studied was revealed, which indicates that electrical conductivity is very sensitive to the structure of the filler phase in the polymer matrix. In general, it was established that the properties, especially the electrical conductivity, of the composites studied strongly depends on the size, shape, and oxidative treatment of CP and PET filler particles, and that the CP coke of appropriately small particle sizes and flake shape has significant potential as a conductive filler for polymer composites.

## 1. Introduction

Technological progress is stimulated by the development of new types of structural materials, the improvement and expansion of the properties of existing materials, or their application in new areas. Polymer composites are one of the most common types of structural materials, and they have gained popularity due to the beneficial combination of properties of both the polymer matrix and the filler, as well as the improvement of the properties of the polymer part due to the reinforcing effect of the filler. Many options for selecting the polymer matrices of composites and various types of fillers, differing not only in physical nature but also in geometric parameters, and various methods for producing composites make it possible to obtain polymer composites with a wide range of electrical, thermal, and mechanical characteristics, which, in turn, allows satisfying the most diverse requirements in the areas where they can be applied.

Among many different types of fillers, the carbon ones are worthy of special notice because they can have a positive impact on the physical and chemical properties of polymer composites [1,2,3]. Over the last few decades, polymer–carbon composites have gained wide popularity in scientific and industrial societies. As a result, the methods for their production were continuously improved [4,5,6,7,8], and the scope of their application expanded [9,10,11,12,13].

Commonly used carbon fillers are carbon fibers, carbon black, and graphite. It was found that carbon fibers significantly enhance the mechanical strength of composites [10,11] and the carbon black additive improves their thermal properties [8,9], while graphite has a positive impact on the mechanical, thermal, and electrical properties of composites [2,12,14]. Less frequently, the fillers based on fossil coals, both raw coals and processed coal products, were used to reinforce polymer matrices [2,15,16,17,18,19,20,21]. Research conducted in this area has shown a beneficial effect of carbon particles on the crosslinking process [18] and on the mechanical properties of polymer composites, including tensile and flexural strength, elongation at break, and elasticity modulus [15,16,17]. The effect of a coal-based carbon filler, resulting in the improvement of the hydrophobic and electrostatic properties of the composite in comparison to a pure polymer matrix, was also noted [19,21]. In general, it has been shown that the final properties of polymer–carbon composites, including internal structure [20] and geometrical parameters of the filler’s particles [3,22,23], depend on the amount and type of filler used.

In the last few years, interest in carbon micro- and nanofillers like carbon black, carbon nanofibers, and nanotubes [3,12], fullerenes [24], CQD [13], graphenic structures [3,14], or thermally expanded graphite [25], rapidly increased. These fillers improve the mechanical properties of carbon–polymer nanocomposites and, importantly, they may cause polymer composites to become electrically or thermally conductive [3,24]. The great interest in conductive carbon micro- and nanofillers is, among others, due to the fact that when the content of their particles in the polymer matrix is above the critical concentration (*φ_c_*—percolation threshold), the resulting composite becomes electrically conductive since a conductive network of filler particles is formed in the volume of the polymer [26]. Thus, the transition from an insulator, a polymer matrix, to a conductor, a formed polymer-based composite filled with carbon particles, is realized.

One of the significant factors that affect the value of the composite percolation threshold is the dispersion system of filler particles in a matrix. If the distribution of filler particles in a polymer matrix is random, then the value of the percolation threshold is quite high. For example, for a composite of PMMA with carbon fibers [26], the *φ_c_* value can be in the concentration range of 20–30 vol.%. For a polyethylene-based composite filled with graphite, *φ_c_* is 14.7 wt.% [27]. And for carbon black-filled composites, *φ_c_* is usually more than 7.0 vol.% [28,29]. When the filler particles are distributed in the matrix in an orderly manner, such composites are characterized by much lower *φ_c_* values [30,31]. Then, for carbon black, the average value of *φ_c_* is in the range of 1–5 vol.% [32,33,34], while for CNT and graphene, the percolation thresholds are less than 1.0% by volume. Winey et al. [35] showed that a composite based on polystyrene filled with single-walled nanotubes (SWCNTs) has a percolation threshold of 0.045 wt.%, while Lisunova et al. [36], for UHMWPE-based composites containing multi-walled nanotubes (MWCNTs), reported values of *φ_c_* = 0.04–0.07 vol.%. In other studies [37], the authors found that a composite based on epoxy resin, in which the nanotubes were oriented under the influence of an external electric field, has a value of *φ_c_* = 0.005 wt.%. Studies of PE-based composites with graphene nanoflakes showed a percolation threshold of *φ_c_* = 0.21 vol.% [38]. An introduction of carbon fillers into a polymer matrix not only improves the electrical and thermal conductivity of composites [38,39,40] but also affects their mechanical and thermal properties as well as the processing parameters [41,42,43]. In general, there is a tendency that with increasing concentration of carbon filler in the composite, its modulus of elasticity, as well as the tensile and bending strength increase, and the elongation at break decreases [15,16,44].

Despite many advantages of polymer composites with carbon fillers, especially with graphene materials, the range of their applications in the industry is limited due to the high costs of production of these fillers, which significantly increases the final price of the products. One of the possible solutions to this problem is the creation of new types of carbon fillers, e.g., based on coals and their modifications [2]. Previous studies of the authors have shown that this type of filler, e.g., anthracite microfiller, may have similar properties to the well-tested carbon black, CNT, and graphene structures, enough for many applications, whereas the cost of their production is lower [20,38,45].

Although the number of articles devoted to the study of alternative carbon materials that could replace nanotubes or graphene as fillers in polymer composites is constantly growing, this topic still has a lot of development potential. Therefore, the purpose of this work was to investigate the conductive, thermophysical, and mechanical properties of polymer composites based on thermoplastic UHMWPE filled with carbon materials obtained in the process of carbonization of petroleum pitch (PET) and coal pitch (CP). The electrical conductivity of these composites was compared with polymer composites containing well-known carbon micro- and nanofillers, such as carbon black, CNTs, graphene, and anthracite. The influence of the above-mentioned carbon nano- and microfillers on the electrical properties of their composites has been studied and described by the authors earlier [38,45].

## 2. Experimental

### 2.1. Materials

The composites for this study were based on ultrahigh molecular weight polyethylene (UHMWPE) Hostalen GUR, type GHR 8110, produced by Hoechst AG (Frankfurt, Germany) in a powdered form. The polymer density is equal to 0.93 g/cm^3^ and the melting temperature is 137 °C. UHMWPE in the form of powder was sequentially sieved through a laboratory sieve shaker and the fraction with average particle diameters of 100 µm was used.

As fillers for the composites, two different carbon materials were used, i.e., coal pitch coke (CP) and petroleum pitch coke (PET), both obtained in a pyrolysis process, at a temperature of ~ 1200 °C in an inert atmosphere. Two different particle fractions of raw CP and PET cokes were used. Individual grain fractions were prepared by grinding the coke in a ball mill and sieving it through sieves with a mesh size of 200 and 50 µm. In the case of sieving with a mesh size of 200 µm, most particles obtained were below 100 µm in size, while very fine particles of around 2 µm in size predominantly passed through a 50 µm sieve. The designations of composites with fillers of different particle sizes are given in Table 1.

Additionally, both cokes oxidized by the modified Hummers method were also used as fillers. The oxidation procedure was carried out in several stages. Firstly, the coke grains with a size < 50 µm were ground with sodium chloride, mixed with hot distilled water, and sonicated in an ultrasonic bath. Then, the mixture was filtered and washed with hot water to remove the chloride ions. The filtered and dried coke powder was mixed with H_2_SO_4_ and H_3_PO_4_ and then cooled to 10 °C. Next, KMnO_4_ was added to the mixture, and after attaining a greenish color, it was slowly heated to 45 °C. The reaction proceeded for 24 h, until the color of the slurry changed from greenish to brown. Then, the slurry was cooled to room temperature and poured onto ice with H_2_O_2_ (30%). Such obtained oxidized coke was purified with HCl solution, water, ethanol, and ether, and, next, it was dried at 50 °C. A detailed description of the oxidation procedure was previously provided by Kumanek et al. [46]. 

All carbon materials used as fillers in this study have layered structures, so they have the potential to be an alternative to graphene fillers.

### 2.2. Preparation of Composites

Polymer–carbon composites were prepared according to the procedure described previously by Mamunya et al. [45], where a scheme for the formation of a segregated structure was presented. UHMWPE and filler powders in a given ratio were thoroughly mixed in a mortar, whereby the carbon fillers electrostatically covered the surface of polymer particles. Then, the mixture was pressed in a closed steel mold at a temperature of 160 °C and a pressure of 20 MPa. The samples for measurements were in the form of discs with a diameter of 30 mm and a thickness of ~1 mm. Such a method of composite preparation led to the formation of a segregated structure of the fillers, which creates a conductive framework in the polymer matrix. 

The composites with PET and CP coke fillers used in this study are listed in Table 1. 

For the analysis of thermo-mechanical properties, crystallinity degree, and Young’s modulus, composites with 10 vol.% of fillers were used. In the case of electrical conductivity, composites with several different contents of fillers in the range of 0.0 to 10.0 vol.% were used to determine the percolation threshold.

### 2.3. Methods for Filler Investigation 

Elemental analysis of initial PET and CP cokes was performed using a Carlo Erba 1106 CHN analyzer (Marlton, NJ, USA) and oxygen content was determined by the difference. The physical and chemical structure of CP and PET fillers were studied by microscopic (SEM-EDS), spectroscopic (Raman, FTIR), and X-ray diffraction (XRD) methods. Morphological studies of coke particles were performed by scanning electron microscopy using a FEI Quanta 250 FEG SEM (Thermo Scientific, Waltham, MA, USA) equipped with a secondary electron detector in a high vacuum mode with an acceleration voltage of 15 kV. The SEM coupled with an energy dispersive X-ray spectrometer (EDAX GENESIS XM 2i, EDAX, Berwyn, PA, USA) was used to determine the content of basic elements (C, O) in the samples investigated. The structural order of the carbon skeleton of PET and CP samples was characterized by Raman spectroscopy and X-ray diffraction. Raman spectra were recorded using a Witec Alpha M300C spectrometer (Oxford Instruments, High Wycombe, UK) with a Nd-YAG laser beam operated with an excitation wavelength of 532 nm and a laser power of 50 mW. For each sample, approximately 7–10 points were analyzed to obtain an insight into the sample’s structural homogeneity. The ratios of the areas of the D and G bands (D/G), estimating the order of the carbon sheets in the cokes’ structures, were determined using Witec Project 4.1 software (Oxford Instruments, High Wycombe, UK). XRD analysis was performed using a diffractometer D8 Advance (Bruker AXS, Karlsruhe, Germany) with CuKα radiation and a LYNXEYE XE-T detector (Bruker AXS, Karlsruhe, Germany). Diffraction patterns in the range of 10–90° 2θ were obtained for all samples. The mean interlayer spacing, d_002_, was evaluated from the position of the (002) peak by applying Bragg’s equation. The mean crystallite sizes, L_c_ and L_a_, were calculated from the (002) and (110) peaks, respectively, using the Scherrer formula. The chemical structure of carbon fillers was determined by FTIR spectroscopy. IR spectra of KBr pellets with coke samples were recorded using an FTIR ATR Nicolet 6700 (Thermo Scientific, Waltham, MA, USA) spectrometer in transmission mode with a resolution of 2 cm^−1^ with 20 scans in the range from 4000 to 400 cm^−1^. The KBr pellets were covered by diluted ethanol solution with coke material and heated to remove the solvent. 

### 2.4. Methods for the Studies of Composite Morphology 

To study the morphology of the composites, 10–20 μm thick sections were prepared with a microtome. The observation of the microstructure of the composites was carried out with a Primo Star optical microscope (Carl Zeiss, Weimar, Germany). Additionally, to show the dispersion and adhesion of filler particles to the polymer matrix, raw fractures of the composites obtained by breaking them in liquid nitrogen were studied by SEM Quanta 250 FEG (Thermo Scientific, Waltham, MA, USA) in low vacuum mode and 10 kV acceleration voltage, using a low-voltage high-contrast backscattered electron detector (Thermo Scientific, Waltham, MA, USA). 

### 2.5. Thermomechanical Analysis

Thermomechanical studies were carried out using a TMA Q400 EM thermomechanical analyzer (TA Instruments, New Castle, DE, USA) in penetration mode with a 0.5 MPa load on the sample in the temperature range of 20–180 °C (the value of penetration of the probe into the sample is denoted as deformation in the text). Composite samples measuring 7 mm × 7 mm and being 1 mm thick, cut from the disks obtained after pressing (see Section 2.2), were used for TMA studies. The samples were placed on a quartz stand and pressed on top by a quartz probe having a tip diameter of 1.2 mm.

The Young’s modulus *E* (MPa) was determined in the mechanical tests using the compression mode of the TMA device by increasing the load with a velocity 0.10 N/min on the sample (stress) and recording the deformation (strain) at a temperature of 20 °C. The *E* value was calculated from the slope of the linear stress/strain ratio. The scattering of *E* values when measuring 5 identical composite samples was within 3%.

### 2.6. DSC Studies

The melting and crystallization behavior of UHMWPE and its composites with PET and CP fillers was studied using a differential scanning calorimeter (TA Instruments DSC Q2000) under a nitrogen atmosphere. Samples were heated from room temperature to 200 °C at a rate of 20 °C/min. After melting, the samples were held in the DSC chamber for 5 min before being cooled to room temperature at a rate of 20 °C/min. From these data, the crystallization temperature (T_c_) and the crystallization enthalpy (Δ*H_c_*) were determined. After completing the first complex melt–crystallization thermograms, samples were heated in the second cycle from 20 °C to 200 °C. The melting temperature (T_m_) and the melting enthalpy (Δ*H_m_*) were determined in accordance with the ISO 11357-3 standard [47]. 

The degree of crystallinity (*X_c_* (%)) of pure UHMWPE and its composites was calculated using Equation (1):

(1) $$X_{c}(\%)=\frac{\Delta H_{m}}{\Delta H_{m}^{o}\cdot (1-\varphi )}\cdot 100$$

where Δ*H_m_* (J/g) is the experimental melting enthalpy value of the sample obtained in the second heating DSC cycle, ϕ is the mass fraction of the filler, and ${\Delta H}_{m}^{{}^{\circ}}$ is the melting enthalpy of 100% crystalline UHMWPE (theoretical value of 293 J/g) [48].

### 2.7. Measurement of Conductivity

The conductivity of the composites was studied by the two-electrode method on direct current (DC). The sample resistance was measured with an E16-13A teraohmmeter (Radiotechnika, Riga, Latvia) at a voltage of 100 V on the electrodes. A composite sample of 30 mm in diameter was placed between two steel electrodes coated with gold on the surface that is in contact with the sample. The measuring electrode had a diameter of 25 mm. The volume conductivity of the material was calculated by the following formula:

(2) $$\sigma =R\frac{l}{S}$$

where *R* is the resistance of the sample, *l* is its thickness, and *S* is the area of the electrode. The conductivity values were averaged from measurements of three samples for each composite. The scatter of resistance measurements from the measurements of three samples was within 10%.

## 3. Results 

### 3.1. Characteristics of Carbon Fillers 

**Figure 1:**
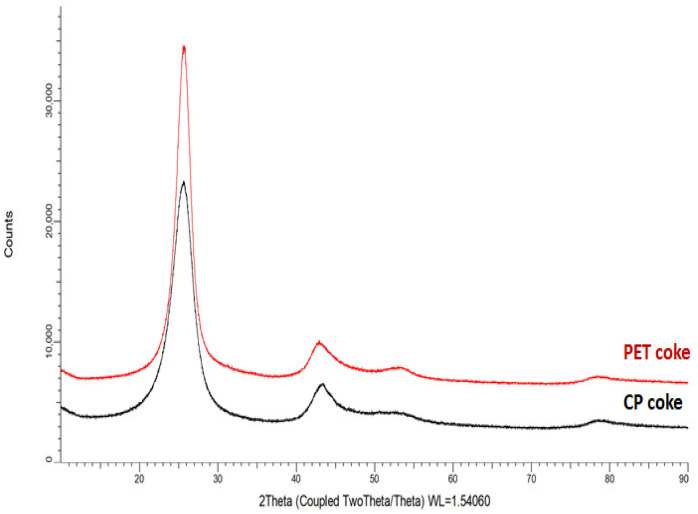
XRD patterns of carbon fillers.

Elemental analysis (Table 2) as well as XRD (Table 3, Figure 1) and FTIR (Table 4) results showed chemical and structural similarity of both CP and PET cokes. However, the FTIR analysis revealed that the CP coke’s chemical structure is more aromatic which corresponds to its higher true density. On the other hand, some aliphatic groups are presented in PET coke, in contrast to CP coke. 

**Figure 2:**
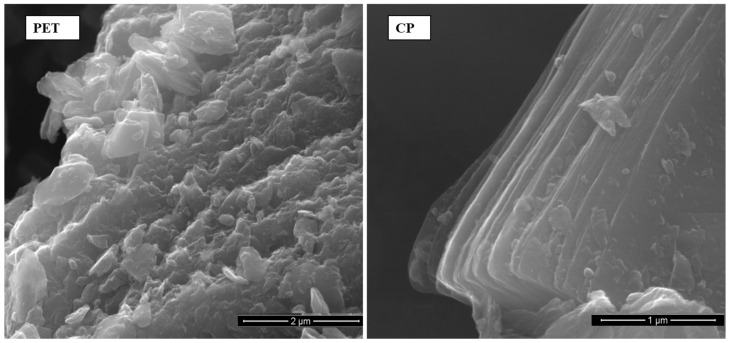
SEM images of raw carbon materials, PET and CP cokes, used as composite fillers.

SEM observation of coke particle morphology showed that CP coke has more distinct layered microstructure compared to PET coke (Figure 2). 

**Figure 3:**
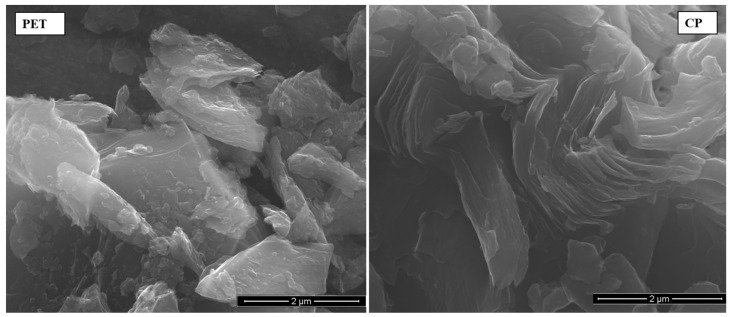
SEM images of raw, deeply ground PET and CP coke grains.

It was also found that during grinding the PET coke was broken down into rather irregular solid crumbs, while the CP coke grains disintegrated mainly along carbon planes giving flake-shaped particles (Figure 3). It can also be seen that the particle size of PET is on average 2 μm whereas flake-shaped CP particles are approximately the same size, but are interconnected. Perhaps this explains the better mechanical and electrical properties of the CP-1 composite (see Section 3.2.4 and Section 3.2.5).

**Figure 4:**
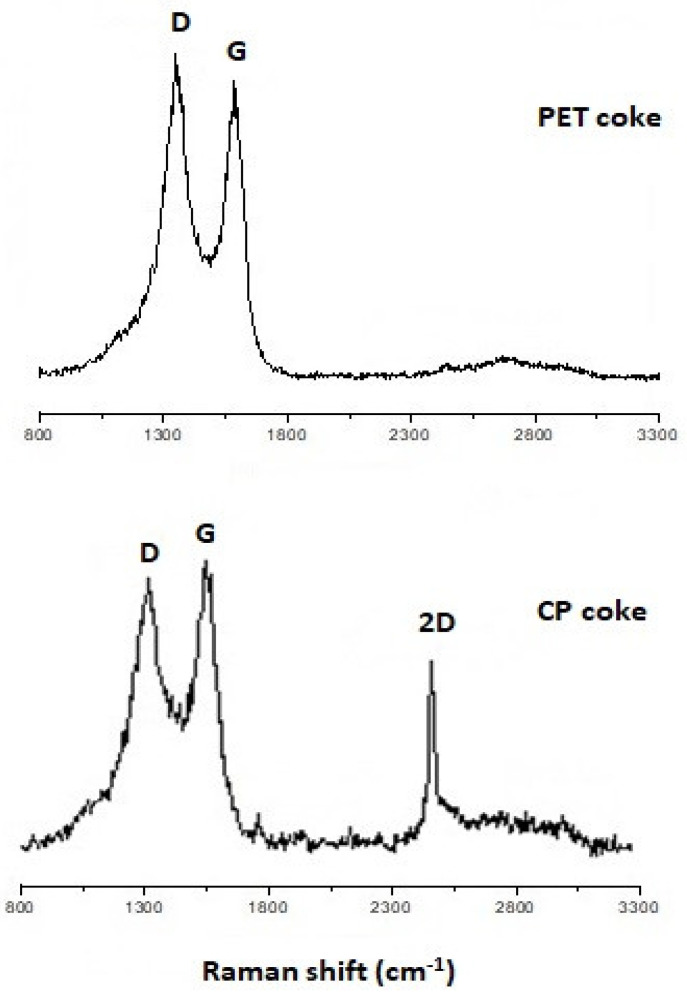
Raman spectra of PET and CP cokes.

Raman spectroscopic analyses confirmed the previous findings obtained by other methods. In Raman spectra of both fillers, evident bands typical for carbon materials were observed (Figure 4). The G bands occurring at 1584 cm^−1^ and 1586 cm^−1^ for the PET and CP cokes, respectively, were attributed to the E2g of polyaromatic C-C structures and the D bands at 1355 cm^−1^ (PET) and 1349 cm^−1^ (CP) associated with disordered, sp^3^-hybridized carbon, were attributed to in-plane defects in graphene sheets or occurrence of heteroatoms [56]. These results showed the similarity of the structural order of PET and CP samples with the domination of less ordered structures in both cokes. However, the D-band to G-band area ratio (A_D_/A_G_) of 2.140 and 2.061 for PET and CP cokes, respectively, indicates a slightly better order in the CP structure. This is also confirmed by the presence of a sharp and narrow 2D band at ~2450 cm^−1^ in the spectrum of CP coke, suggesting the occurrence of graphene structures with a high degree of structural order [57,58]. This band is not visible in the spectrum of the PET sample.

The results of SEM-EDS analyses of both grain fractions of CP and PET cokes, as well as of these cokes oxidized by the Hummers method are presented in Table 5. As expected, for both CP and PET coke the oxygen content is higher in the finer grain fraction. In the case of oxidized cokes, it is seen that the oxidation process was much stronger in CP coke compared to PET coke, which is manifested in more than twice the oxygen content in it. This is probably due to the flake shape of the fine CP particles, which have a larger surface area than irregular PET crumbs and are therefore more oxidizable. It is also clearly visible that the C/O ratio for the CP-Hum sample is much lower than for the other coke samples, which significantly influenced the properties of the composites with this filler.

### 3.2. Characteristics of Composites

#### 3.2.1. Structure of Composites

In the process of pressing, the polymer granules are melted and joined together, forming a continuous polymer matrix. In this case, the filler particles remain localized at the boundary between the polymer granules and form a framework in the polymer matrix. Thus, a segregated structure of the polymer composite is developed. The local filler concentration in the framework wall is much higher than the average concentration calculated for the entire sample volume, which determines the electrical properties of the composite [38,59].

**Figure 5:**
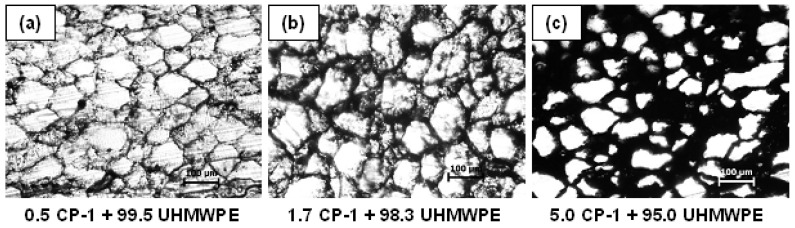
Optical microscopic images of the structure of the UHMWPE-based CP-1 composite with different filler concentrations: (**a**) 0.5 vol.%; (**b**) 1.7 vol.%; (**c**) 5.0 vol.%. Particle size distribution based on optical microscopy analysis: size (μm)/fraction (%)—50/6, 60/7, 80/12, 90/10, 100/17, 120/6, 140/7, 160/3, 170/2.

Figure 5 shows the optical microscopic images of the structure of the CP-1 composite with different filler concentrations: below the percolation threshold (a), at the percolation threshold (b), and well above the percolation threshold (c). It is seen from the photos that the polymer granules are surrounded by a framework formed from the filler particles and the thickness of the framework increases with increasing filler concentration.

**Figure 6:**
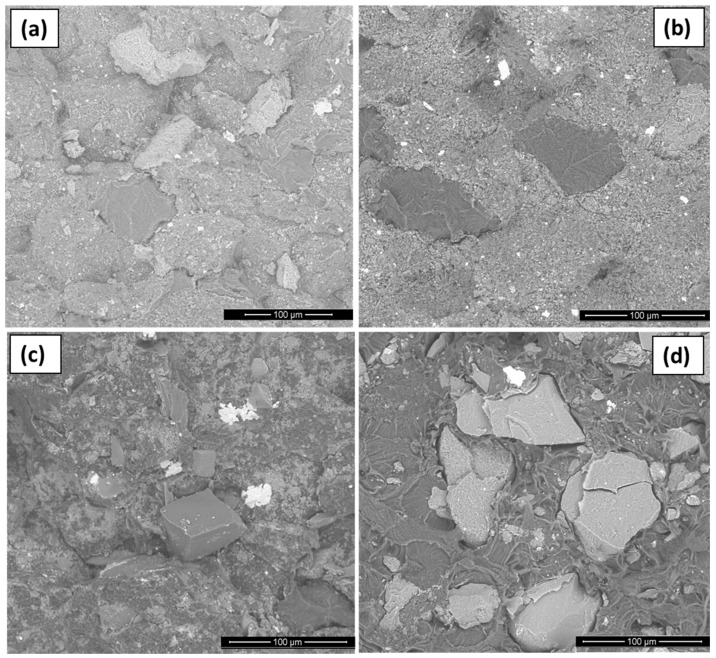
SEM images of fragments of the composites PET-2 (**a**) and CP-2 (**b**) with raw coke grains as well as PET-Hum (**c**) and CP-Hum (**d**) with oxidized coke particles.

SEM analyses of composite fracture morphology showed a large particle size distribution of both PET-2 and CP-2 fillers and also revealed the dispersion and adhesion of filler particles to the polymer matrix, good in the case of raw coke grains (Figure 6a,b) and worse in the case of oxidized coke particles (Figure 6c,d). Apparently, as a consequence, the composite CP-Hum has the worst mechanical and electrical characteristics (see Section 3.2.4 and Section 3.2.5). In UHMWPE–carbon filler composites there are no chemical interactions due to the absence of reactive groups, so only physical interactions exist. The influence of different physical interactions on the properties of composites is described in the reviews [60,61].

#### 3.2.2. DSC Study of Composites

**Figure 7:**
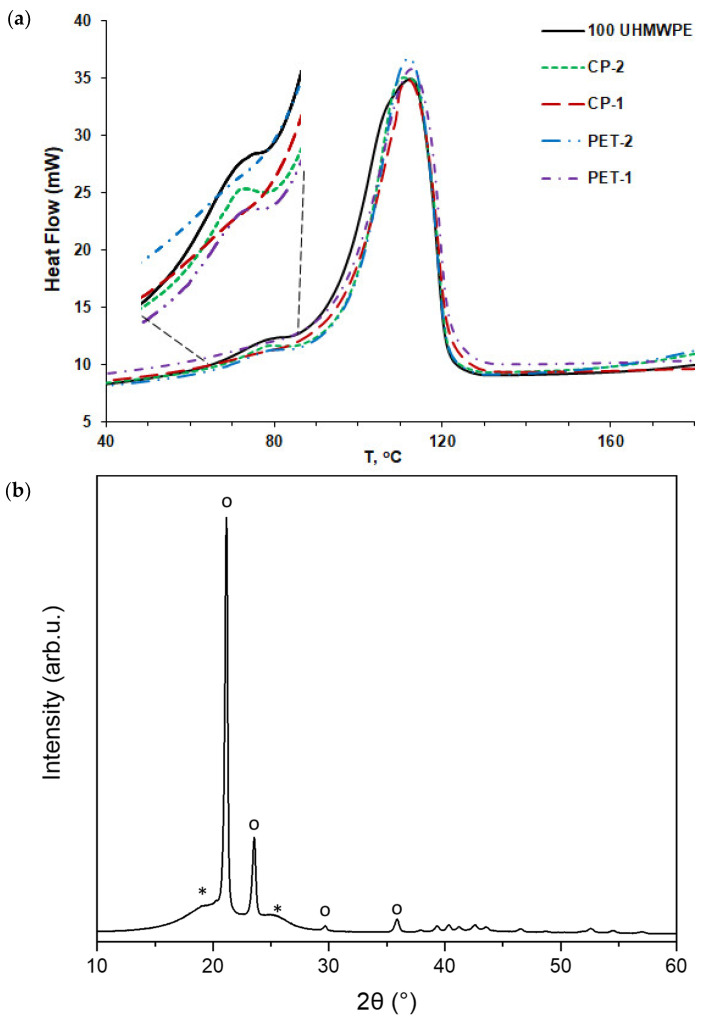
(**a**) Crystallization peaks of pure UHMEPE and UHMWPE-based composites filled with various grain fractions of PET and CP cokes. The insert shows the behavior of the crystallization curves in the low temperature range of 60–90 °C; (**b**) XRD pattern typical for the composites with coke particles < 200 µm; o—orthorhombic phase of polyethylene, *—monoclinic phase of polyethylene.

**Figure 8:**
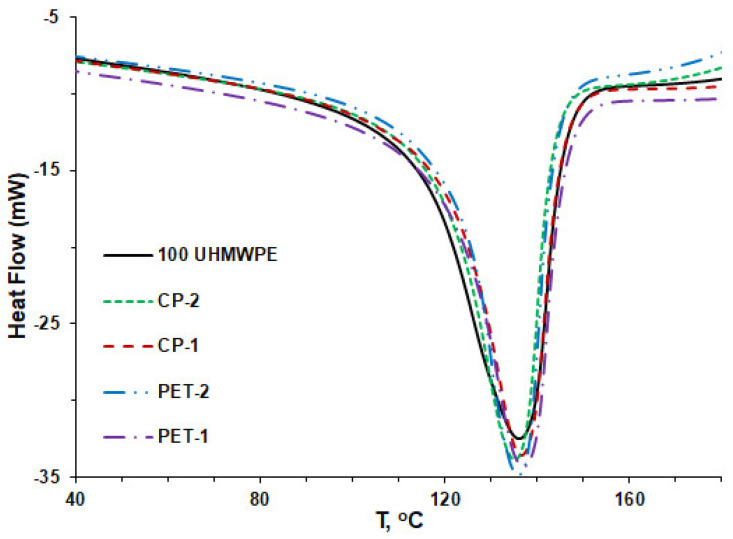
Melting peaks of pure UHMWPE and UHMWPE-based composites filled with various grain fractions of PET and CP cokes (second run).

The effect of the introduction of PET or CP coke grains on the crystallization and melting behavior of UHMWPE in composites was studied using non-isothermal DSC measurements. Figure 7 and Figure 8 show the results of DSC studies, namely the peaks corresponding to the crystallization (Figure 7) and melting (Figure 8) for the composites with 10 vol.% coke fillers.

Figure 7a shows the crystallization peaks for pure UHMWPE and for the composites PET-1, PET-2, and CP-1, CP-2. As one can see, the intensity and the temperature of the peaks differ a little depending on the grain fraction of the coke filler in the composite. The curves of pure UHMWPE and the composites CP-2 and PET-2 (with bigger coke particles) have additional low-intensity crystallization peaks at a temperature of about 75 °C. According to the XRD data typically obtained for the composites studied (Figure 7b), this fine peak might correspond to the crystallization of a minor amount of a monoclinic phase of the polymer. This effect is not observed in the composites PET-1 and CP-1 with small filler particle size, most likely due to the restricted effect of smaller coke particles on the crystallization of UHMWPE at lower temperatures, which hinders crystallization of the monoclinic phase. 

**Figure 9:**
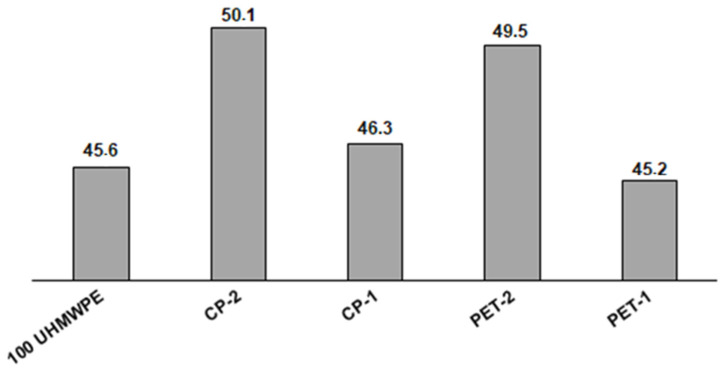
Values of the degree of crystallinity (*X_c_*) for the pure UHMWPE and UHMWPE–coke filler composites.

Figure 8 shows the DSC thermograms of the composites containing various grain fractions of PET and CP cokes in the melting region. As can be seen, the courses of the curves for individual composites are just a little different, the melting point for all composites is 137 ± 1 °C, which is close to the values presented for UHMWPE composites filled with MWCNT [62] and a UHMWPE–boron nitride system [63]. However, the calculation of the crystallinity degree (α) from the curves shows that the difference in this parameter is about 5% between the composites with coke fillers of bigger particle size and smaller particle size. As one can see in Figure 9, the values of the crystallinity degree of the composites PET-1 and CP-1 are similar to pure UHMWPE and lie in the range of 45.2–46.4%. Similar values of the degree of crystallinity for pure UHMWPE were found by Khalil et al. [64], α = 43.1%, as well as in refs. [65,66], α = 40%. In the composites PET-2 and CP-2, containing fillers with bigger particle sizes, the degree of crystallinity of the polymer matrix is higher and varies from 49.5 to 50.1%. 

This phenomenon can be explained by the greater share of finer filler particles in the composites CP-1 and PET-1. The greater content of smaller particles contributes to a tighter coverage of the surface of polyethylene grains and, consequently, to a slightly lower intensity of the melting process and lowering of the degree of crystallinity. 

#### 3.2.3. Thermomechanical Studies of Composites

**Figure 10:**
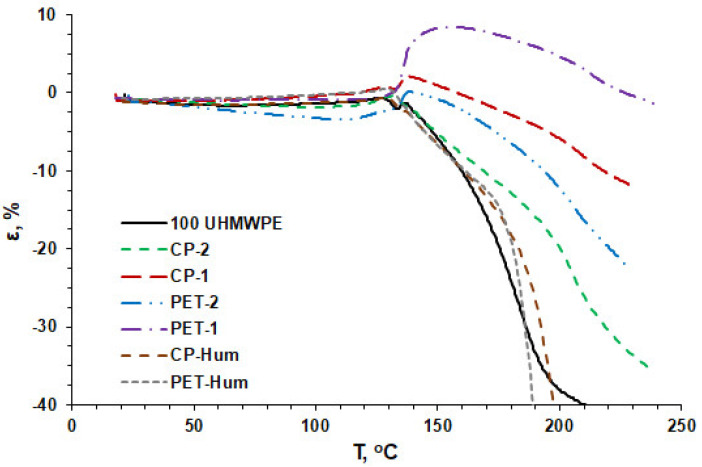
Relative deformation of composites with increasing temperature of the samples.

Figure 10 presents the results of thermomechanical analyses of composites with 10 vol.% of PET and CP cokes. The TMA curves show the values of the relative deformation of the samples depending on the temperature at a constant load of 0.5 MPa. The relative deformation ε was calculated as

(3) $$\varepsilon =\left(L-L_{0}\right)/L_{0}\cdot 100\%$$

where *L*_0_ is the initial sample thickness and *L* is the current sample thickness.

In Figure 10, it is generally seen that when the temperature exceeds 125 °C, the deformation of the samples slightly increases, and sharply decreases just after that, which indicates a transition to the melting region of the composites (T > T_m_). In this case, the deformation is due to the decreasing thickness of the samples, which leads to their negative values. However, for the composite PET-1, an increasing thickness of the sample is observed and the maximum strain reaches ~10% at a temperature of 150–160 °C. 

The behavior of the composite PET-1 is completely different. Starting from a temperature of 130 °C, an increase in the thickness of the sample is observed and the maximum strain, ~10%, is reached at a temperature of 150–160 °C. Such a feature of this composite can be explained by the appearance of outgassing when approaching the temperature of the premelting region, which increases the volume of the composite first in a softened state and, further, in a melted state. 

The curves for CP-Hum and PET-Hum composites are similar to each other and are characterized by a rapid increase in deformation with temperature, which is similar to pure UHMWPE. Presumably, this is the result of poorer adhesion and, consequently, a weak reinforcing effect of oxidized PET and CP coke particles.

#### 3.2.4. Mechanical Analyses of Composites

**Figure 11:**
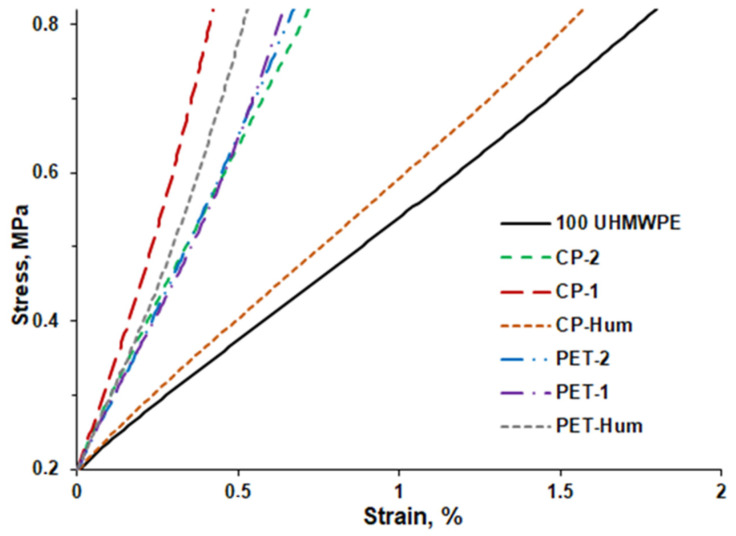
Stress/strain curves for the pure UHMWPE and UHMWPE–coke filler composites.

For the studied composites, the stress/strain dependences were investigated in the stress range of 0–0.6 MPa using the compression mode, which is shown in Figure 11. These curves are similar to those obtained in previous studies for UHMWPE with different carbon fillers [67,68]. As can be seen from the figure, in this stress interval, these dependencies are almost linear and were used to determine the values of Young’s modulus from the slope of the straight lines.

**Figure 12:**
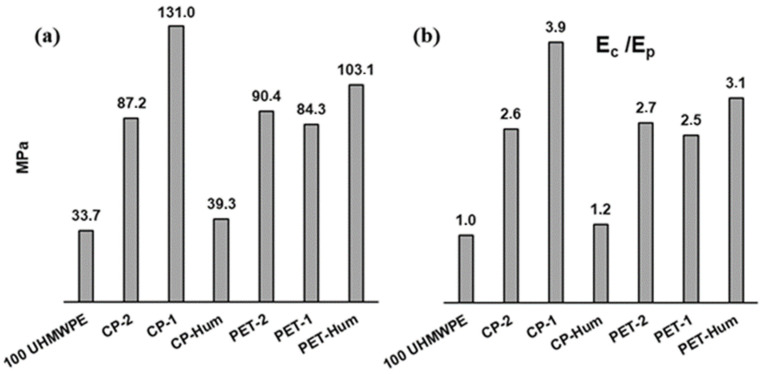
Young’s moduli of pure UHMWPE and its composites with coke fillers (**a**) and normalized Young’s moduli (**b**).

The calculated values of the Young’s modulus for the composites are shown in Figure 12a. As can be seen, the lowest value of Young’s modulus is observed for pure UHMWPE (E = 33.7 MPa), while the highest value is for the composite CP-1 (E = 131.0 MPa). Very different values of the Young’s modulus of pure UHMWPE and its composites can be found in the literature, which may depend on sample processing and test methods (tensile or pressure) [69,70,71,72], as well as for other composite materials with different polymer matrices [24]. 

There is a great variety of Young’s moduli of the composites relative to the modulus of the polymer matrix, *E_c_*/*E_p_* (Figure 12b). As can be seen, the composite with CP-1 demonstrates the greatest increase in the modulus (almost four times), whereas the smallest increase in modulus is observed for the composite with CP-Hum (1.17 time). The increase in the Young’s modulus values of the remaining composites related to pure UHMWPE is similar and within the range of 2.50–3.10. 

The increase in Young’s modulus values after introducing the filler into the polymer matrix is the effect of the formation of a regular segregated structure of the filler when it is localized in the intergranular region of the polymer during the formation of the composite by hot compaction. The more rigid the filler framework that is formed, the higher the Young’s modulus value of the composite. The rigidity of the framework depends on the strength of the contacts between the particles of the filler, as well as on the possible interaction of the filler particles with the polymer matrix. The strength of interactions between the small flake-shaped particles of CP-1 seems to be the highest among the used CP and PET coke fillers, which results in a significantly higher Young’s modulus of the CP-1 composite compared to the other composites. The possible influence of the morphology and size of carbon fillers on the mechanical properties of polymer composites is discussed in ref. [22].

#### 3.2.5. Electrical Conductivity of Composites

**Figure 13:**
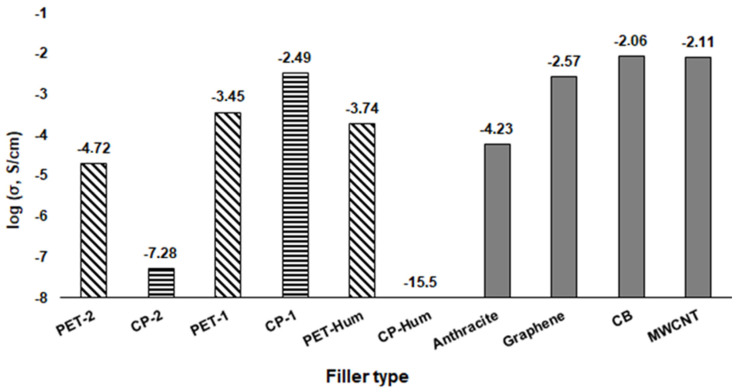
Electrical conductivity of composites based on UHMWPE filled with coke fillers of various grain fractions and with conventional carbon nanofillers. The conductivity values for composites with anthracite and graphene are taken from ref. [38], and with MWCNT from ref. [73].

Figure 13 shows electrical conductivity values for composites containing the coke-based PET and CP fillers and the conventional carbon microfiller (anthracite) and nanofillers (graphene, MWCNT, CB). The concentration of fillers in all cases was 10 vol.%. The highest value of electrical conductivity among the composites with coke fillers was achieved for the CP-1 composite with the small coke particle size. The value of electrical conductivity for this composite is close to the value of conductivity of composites with graphene, MWCNT, and CB and is two decimal orders higher than that of composites with anthracite. 

The electrical conductivity of composites with PET coke filler is higher than that with CP filler in the case of bigger particle sizes (PET-2 and CP-2) and lower in the case of smaller particle sizes (PET-1 and CP-1). For both coke fillers, electrical conductivity of composites increases with decreasing dimensions of filler particles. However, for the composite CP-1, the increase in the conductivity value is much more significant (the difference between composites with PET-1 and CP-1 reaches 3.5 decimal orders). This may be due to the fact that the fine, flake-shaped particles of CP coke contain a number of well-ordered carbon planes similar to graphene structures, which was revealed by the SEM (Figure 2 and Figure 3) and Raman spectroscopy (Figure 4), and are characterized by better conductivity than the more isometric crumbs of PET. Due to this, the CP-1 particles exhibit interconnectivity (Figure 3), which contributes to increased conductivity.

The electrical conductivity of composites with coke fillers oxidized by the Hummer method is completely different depending on the type of coke. For the composite with PET-Hum coke, its electrical conductivity is close to the composite with the raw PET coke of a smaller particle size, whereas the composite with CP-Hum coke is completely non-conductive. This difference is probably the result of different degrees of these fillers’ oxidation, which is significantly greater in the case of the CP-Hum filler. From previous studies, it is known that the oxidation of graphene planes reduces their electrical conductivity. The most important is the C:O ratio in graphene oxide. When it is 2:1 or lower the GO becomes nonconductive [3,59,74]. Considering the SEM-EDS results listed in Table 5, one can see that in the CP-Hum sample, the C:O ratio is <2:1; therefore, this filler is not conductive, resulting in a lack of its composite conductivity. In addition, Figure 6d shows poor adhesive contact of the CP-Hum coke particles with the polymer matrix, which also contributes to a decrease in the conductivity of the composite with CP-Hum. In general, carbon fillers prepared by the carbonization of various materials can exhibit high conductivity depending on the initial product and the size and shape of the resulting carbon particles. For example, composites based on poly(3-hydroxybutyrate-co-3-hydroxyvalerate) filled with biocarbons, which were prepared from wood dust and cellulose fibers, had a conductivity value of 0.7 S/cm [75]. 

Table 6 presents the electrical conductivity of composites of different polymer matrices and carbon fillers reported in the literature to compare them with UHMWPE–PET and UHMWPE–CP composites.

**Figure 14:**
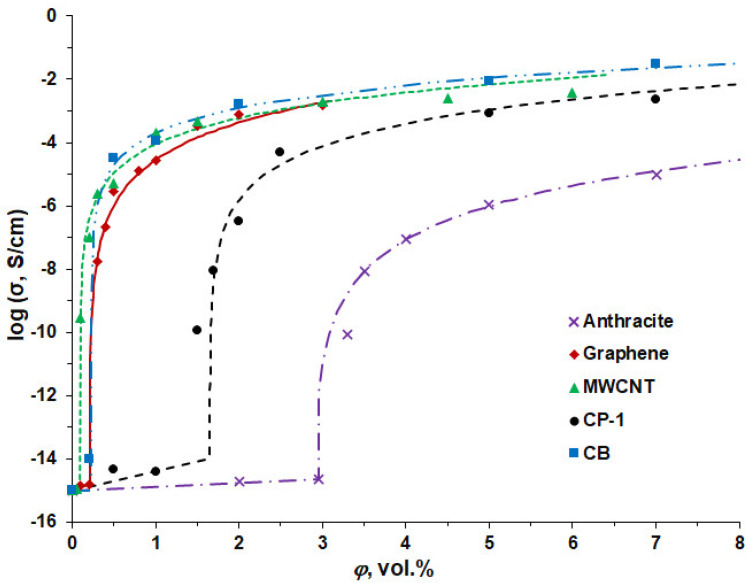
Concentration dependence of the electrical conductivity of composites with different types of carbon fillers. Points are experimental values, and solid lines are calculated by the percolation equation (in the region *φ* > *φ_c_*). Percolation curves for anthracite, graphene, and MWCNT fillers are taken from our previous studies [38,73].

Figure 14 shows the concentration dependence of the conductivity of the composite CP-1, as well as previously studied composites with anthracite microfiller, and graphene, MWCNT, or carbon black fillers [38,73].

To describe the relationship between the filler concentrations and the electrical conductivity of the composites, the percolation equation was used [77], which enabled us to obtain the values of the percolation parameters *φ_c_*, *t*, *σ_m_* given in Table 7:

(4) $$\sigma =\sigma_{m}\cdot {\left(\varphi -\varphi_{c}\right)}^{t}$$

where *σ_m_* is the parameter of conductivity related to the filler phase; *φ* is the concentration of the filler in the composite; *φ_c_* is the value of the percolation threshold; and *t* is the critical index.

For the composite CP-1, the percolation threshold value *φ_c_* is 1.6 vol.%, which is almost half than for the composite with an anthracite microfiller (2.9 vol.%) but is much higher than for the other conventional carbon fillers. This can be explained by the dimensional factor, since graphene, MWCNT, and CB are nanofillers, for which the percolation threshold is always lower than for microfillers [78,79]. It was shown in [80] that a PP–graphene composite with a segregated filler structure has a percolation threshold value of 0.04 vol.%. At the same time, Zhao et al. [76] found that UHMWPE composites with the segregated filler structure of reduced graphene oxide, natural graphite, and carbon nanofibers have the percolation threshold values in the range of 1–6 phr. 

The theoretical value of a critical exponent for systems with a statistical, random distribution of the conductive inclusions in nonconductive media is *t* = 2. Higher *t* values for the studied composites are due to the nonstatistical, ordered filler distribution in the polymer matrix in segregated systems. For the composite CP-1, the parameter *σ_m_*, which, with some approximation, can be considered as the value of the filler conductivity, has a value an order of magnitude higher than the conductivity of anthracite and approximately equal to the conductivity of MWCNT, but an order of magnitude lower than graphene. 

**Figure 15:**
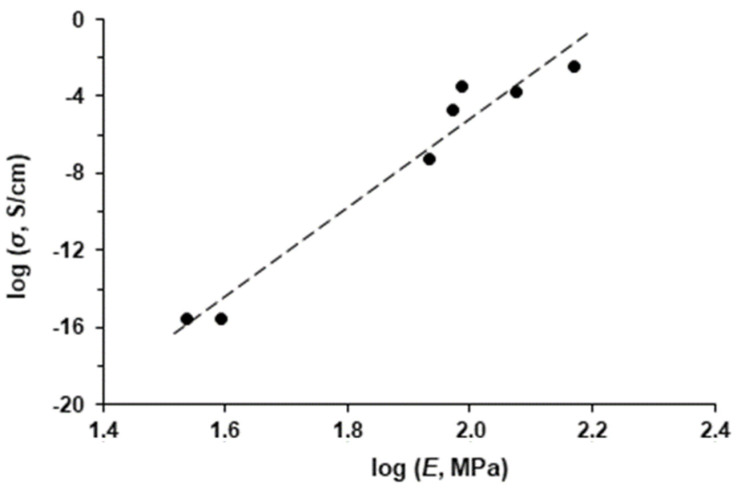
Correlation between Young’s modulus (*E*) and electrical conductivity (σ) of UHMWPE–coke filler composites.

Good contact between filler particles affects both the elastic properties of a composite and its electrical conductivity. Thus, a correlation between the Young’s modulus and the conductivity of the composites can be expected. The relationship between the Young’s modulus and the electrical conductivity (on a logarithmic scale) of the samples studied is shown in Figure 15.

As can be seen, there is a linear relationship between log *E* and log *σ*. From the slope of this, it follows that the change in the Young’s modulus by 1.4 times corresponds to the change in the electrical conductivity by four orders of magnitude. From this, it can be concluded that the electrical conductivity of a composite is very sensitive to the structure of the conductive phase formed by the filler particles in the polymer matrix.

## 4. Discussion and Conclusions

The composites based on ultrahigh molecular weight polyethylene (UHMWPE) containing microfillers based on coal pitch (CP) and petroleum pitch (PET) cokes of different particle sizes were studied in terms of their structure as well as thermal, mechanical, and electrical properties. It was found that the structure and properties of the composites studied strongly depend on the type, size, shape, and treatment of the filler particles. 

Two types of the composites contained filler particles of two different sizes, about 2 μm (CP-1, PET-1) and about 100 μm (CP-2, PET-2). CP-1 and PET-1 particles obtained by grinding of bigger grains, gain new properties, a layered microstructure becomes apparent in them and they acquire a flake-shaped form, especially the CP coke, which provides higher electrical conductivity compared to the composites, having larger particles of fillers (CP-2, PET-2). 

Using microscopic (SEM-EDS), spectroscopic (Raman, FTIR), and X-ray diffraction (XRD) methods, the characteristics of carbon fillers were studied. Here, the chemical and structural similarity of both CP and PET cokes was indicated. On the other hand, the FTIR analysis revealed that the CP coke’s chemical structure is more aromatic, which corresponds to its higher true density, and some aliphatic groups are present in the PET coke in contrast to CP coke. The Raman spectroscopy analysis showed the similarity in the structural order of PET and CP samples with a predominance of a less ordered structure in both cokes, whereby a slightly better ordering of the CP structure was indicated. The 2D band in the spectrum of the CP coke suggests the occurrence of layered graphene structures with a high degree of structural order, whereas this band is not visible in the spectrum of the PET sample. The SEM analysis revealed that during grinding, the PET coke was broken down into rather irregular solid crumbs, while the CP coke grains disintegrated mainly along carbon planes giving flake-shaped particles with an average size of 2 μm, which are interconnected.

The fine, flake-shaped particles of the CP-1 coke contain a number of well-ordered carbon planes similar to graphene structures and are characterized by better conductivity than the more isometric crumbs of PET-1. As a result, the CP-1 particles exhibit interconnectivity, which contributes to increased conductivity. The conductivity of the CP-1 composite is the highest among the composites studied (3.2 × 10^−3^ S/cm) and is at the level of composites with known carbon nanofillers: graphene (2.7 × 10^−3^ S/cm), carbon black (8.7 × 10^−3^ S/cm), and CNT (7.7 × 10^−3^ S/cm). The other composites have lower conductivity in the range of 5.2 × 10^−8^–3.5 × 10^−4^ S/cm.

The studied composites demonstrate the percolation character of conductivity. Calculation of the percolation parameters according to the percolation equation shows that the percolation threshold *φ_c_* of the CP-1 composite is 1.6 vol.%, which is almost half than for the composite with an anthracite microfiller (2.9 vol.%) but much higher than that for the other conventional carbon nanofillers. The parameter *σ_m_* of the percolation equation, which is related to the conductivity of the filler, has a value an order of magnitude higher than the conductivity of anthracite and approximately equal to the conductivity of MWCNT, but an order of magnitude lower than that of graphene. 

Mechanical measurements using the TMA method provided stress-strain relationships, from which the Young’s modulus could be calculated. The highest value of the Young’s modulus *E* was found for the composite CP-1, which is due to a more rigid filler framework in the polymer matrix. The Young’s moduli correlate with the conductivity values *σ* of the composites. The linear relationship between log *E* and log *σ* shows that the change in Young’s modulus by 1.4 times corresponds to the change in electrical conductivity by four orders of magnitude. This is evidence for the electrical conductivity of a composite being very sensitive to the structure of the conductive phase formed by the filler particles in the polymer matrix.

The comparison of mechanical and electrical properties of the composites with different size and shape of particles of the PET and CP coke fillers indicates their relationship, whereby the flake shape of the CP coke particles seems to have a greater impact on the values of electrical conductivity and Young’s modulus of the composites than the size of the filler particles themselves.

In general, the results of this study showed the significant potential of coal pitch coke (CP) of appropriately small particle size and flake shape as a conductive filler for polymer composites. Composites with this filler have properties similar to composites with carbon nanofillers such as graphene, carbon black, and MWCNT.

## Figures and Tables

**Table 1:** Types and labels of the composites used in this study.

Polymer Matrix	Coke Filler	Composite Label
UHMWPE	PET < 100 µm	PET-2
	PET~2 µm	PET-1
	PET oxidized	PET-Hum
	CP < 100 µm	CP-2
	CP~2 µm	CP-1
	CP oxidized	CP-Hum

**Table 2:** Chemical composition and true density of initial PET and CP cokes.

Element (wt.%)	PET	CP
Carbon	93.70	95.98
Hydrogen	traces	traces
Oxygen nitrogen	0.45	0.29
Oxygen *	5.85	3.73
True density (g/cm^3^)	1.9768	2.0340

* Determined by the difference.

**Table 3:** Structural parameters (nm) of carbon fillers.

	L_a_	L_c_	d_(002)_
PET	73	11	0.348
CP	54	10	0.346

**Table 4:** The results of FTIR analysis of carbon fillers [49,50,51,52,53,54,55].

PET	CP	Interpretation of FTIR Bands
878 cm^−1^	875 cm^−1^	Ortho-substitution of aromatic bending C-H (700–900 cm^−1^) Benzene rings, more hydrophobic than oxygen functional groups (867 cm^−1^)
1237 cm^−1^	-	C-O stratching and O-H bending vibration (1100–1300 cm^−1^) -C-O (1224 cm^−1^) C-O-C (1300–1150 cm^−1^) Aliphatic CHx bending (1100–1300 cm^−1^)
1385 cm^−1^	1385 cm^−1^	COO (1382 cm^−1^) Alkane functional group with medium C-H aliphatic bending (1385 cm^−1^)
-	1447 cm^−1^	C=C aromatics Methylene bending vibration (1432 cm^−1^) Bending of aliphatic C-H band (1450–1370 cm^−1^) C=C stretching of aromatic ring (1431–1505 cm^−1^)
-	1628 cm^−1^	C=O carbonyl group (1700–1600 cm^−1^)
3480 cm^−1^	3480 cm^−1^	NH/OH/COOH (3480 cm^−1^)

**Table 5:** SEM-EDS results of crushed raw and oxidized PET and CP cokes.

Element (wt.%)	PET-2	PET-1	PET Hum	CP-2	CP-1	CP Hum
Carbon	93.34	92.48	84.40	95.55	95.34	62.84
Oxygen	6.46	7.21	15.09	3.83	4.56	34.85
Others	0.20	0.31	0.51	0.62	0.10	2.31
C/O ratio	14.45	12.83	5.60	24.95	20.91	1.80

**Table 6:** Electrical conductivity of various polymer–carbon composites—data from the literature.

Polymer	Filler	Particle Size	Filler Concentration	Electrical Conductivity *σ* (S/cm)	Reference
UHMWPE	NG	-	5 phr	~10^–15^	[76]
	CNT	-	5 phr, 20 phr	2.0 × 10^−4^, 1.03 × 10^−2^	
	rGO	-	5 phr	2.4 × 10^−5^	
UHMWPE	Anthracite	5 μm	20 vol.%	3.0 × 10^−2^	[45]
	Graphene	XY—<10 μm, Z—<3 layers	2 vol.%	1.0 × 10^−2^	
	TEG	cells 5–10 μm, walls 40–80 nm	5 vol.%	2.6 × 10^−2^	
Epoxy	EG	Lateral size < 106 μm	16 mass%	1 × 10^−1^	[3]
	GNP	Lateral size < 14 μm	2 mass%	3.6 × 10^−4^	
		thickness 10–20 nm			
	GO	Avg. lateral area 191.3 μm^2^	2 mass%	10–12	
	rGO	Lateral size ~10 μm	5 mass%	7.8 × 10^−7^	
UHMWPE	Graphene	-	1.5 mass%	~10^−3^	[61]
UHMWPE	Graphene	-	1.0 mass%	~10^−2^	
PP	GO	-	4.9 mass%	3 × 10^−3^	
UHMWPE	rGO	-	4.0 mass%	7.1 × 10^−2^	
PE	Graphene	-	-	2.7 × 10^−3^	[73]
	CB	-	-	8.7 × 10^−3^	
	CNTs	-	-	7.7 × 10^−3^	
poly(3-hydroxybutyrate-co-3-hydroxyvalerate)	Carbonized cellulose	-	20 vol.%	6.9 × 10^−1^	[75]
	Carbonized wood	-	10 vol.%	3.7 × 10^−12^	
UHMWPE	CP coke	~2 µm	10 vol.%	3.2 × 10^−3^	[this work]
	CP coke	<100 µm	10 vol.%	5.2 × 10^−8^	
	PET coke	~2 µm	10 vol.%	3.5 × 10^−4^	

**Table 7:** Percolation parameters of electrical conductivity of the composites with the CP-1 filler and conventional carbon micro- and nanofillers [38,45,73].

Filler	*φ_c_*, vol.%	*t*	*σ_m_*, S/cm
CP-1	1.6	2.9	21
Anthracite	2.9	3.8	2.3
CB	0.23	2.2	8.9
Graphene	0.21	3.3	251
MWCNT	0.09	2.6	17.8

## Data Availability

Data are contained within the article.
